# The Influence of Nutritional Intervention in the Treatment of Hashimoto’s Thyroiditis—A Systematic Review

**DOI:** 10.3390/nu15041041

**Published:** 2023-02-20

**Authors:** Karolina Osowiecka, Joanna Myszkowska-Ryciak

**Affiliations:** 1 Doctoral School, Warsaw University of Life Sciences (WULS), 02-787 Warsaw, Poland; 2 Department of Dietetics, Institute of Human Nutrition Sciences, Warsaw University of Life Sciences (WULS), 02-776 Warsaw, Poland

**Keywords:** Hashimoto’s disease, thyroid, autoimmune disease, nutritional intervention, diet

## Abstract

Diet can be a complementary treatment for Hashimoto’s disease by affecting thyroid function and anti-inflammatory properties. It is still unclear which dietary strategy would be the most beneficial. The aim of this systematic review is to examine all the data currently available in the literature on the effects of nutritional intervention on biochemical parameters (anti-thyroid antibody and thyroid hormones levels) and characteristic symptoms in the course of Hashimoto’s thyroiditis. This systematic review was prepared based on PRISMA guidelines. Articles in PubMed and Scopus databases published up to November 2022 were searched. As a result of the selection, out of 1350 publications, 9 were included for further analysis. The nutritional interventions included the following: elimination of gluten (3 articles) or lactose (1 article), energy restriction with or without excluding selected foods (*n* = 2), consumption of *Nigella sativa* (*n* = 2), or dietary iodine restriction (*n* = 1). The intervention duration ranged from 21 days to 12 months and included individuals with various thyroid function. Of the nine studies, three studies were female only. An improvement was observed during an energy deficit and after the elimination of selected ingredients (e.g., gluten, lactose, or goitrogens), as well as after the intervention of *Nigella sativa*. These interventions improved antibody levels against peroxidase (anti-TPO), (thyrotropin) TSH, and free thyroxine (fT4). No improvement was seen on the iodine-restricted diet. Varied outcomes of analyzed dietary interventions may be due to the heterogeneous thyroid condition, high variability between patients, and differences in habitual intake of critical nutrients (e.g., iodine, selenium, and iron) in different populations. Therefore, there is a great need for further experimental studies to determine whether any nutritional interventions are beneficial in Hashimoto’s disease.

## 1. Introduction

Hashimoto’s thyroiditis (HT), also known as chronic lymphocytic thyroiditis, is one of the autoimmune disorders of the thyroid gland. This disease is more often diagnosed in women than in men (350 vs. 80 per 100 thousand people a year) [1]. In the development of Hashimoto’s disease, genetic factors account for 70–80% of the risk and environmental factors for 20–30% [2]. HT is diagnosed on the basis of the presence of antibodies in the thyroid gland (anti-TPO, i.e., antibodies against thyroid peroxidase and anti-TG, i.e., antibodies against thyroglobulin) [3]. HT is also characterized by the infiltration of lymphocytes into thyroid tissue, which can be observed on ultrasound. Other diagnostic markers recommended for blood tests are TSH (thyrotropic hormone) and fT4 (free thyroxine) [2,3]. Hashimoto’s disease worsens the functioning of the thyroid gland, the hormones of which have multiple effects on other organs and tissues. The symptoms of Hashimoto’s disease include, among others, chronic fatigue, nervousness, mood swings, and gastrointestinal or cardiovascular problems [3]. Treatment includes replacement of thyroid hormones for hypothyroidism associated with HT. The basic pharmacotherapy is levothyroxine [4]. Despite the achievement of euthyroidism (state of normal thyroid hormone levels), the quality of life of some patients is unsatisfactory. This may be because of the impact of autoimmunity, although the results are inconclusive [5,6,7].

The implementation of a diet is a non-invasive method that can provide measurable benefits. The literature emphasizes the fundamental influence of various nutrients. Anti-inflammatory nutrients, such as vitamin D, antioxidants, monounsaturated and polyunsaturated fatty acids, magnesium, and zinc, are important to reduce thyroid inflammation. [8]. Iodine and selenium are involved in the synthesis and metabolism of thyroid hormones [9]. An adequate supply of iron, folic acid, and vitamin B_12_ is also important as a result of frequent anemia and cardiovascular diseases in this group of patients with Hashimoto’s thyroiditis [3]. According to current knowledge, gluten or lactose should be eliminated in the presence of food intolerances or diseases such as celiac disease [9,10,11]. One of the principles of a properly balanced diet is to limit saturated fatty acids, sugars, and refined carbohydrates, which have a pro-inflammatory effect [8,10]. Such an anti-inflammatory diet is represented, among others, by model of the Mediterranean diet. In a study by Ruggeri et al. [12], Hashimoto’s patients who adhered to the principles of the Mediterranean diet had lower oxidative stress parameters, which can have an impact on reducing the inflammatory process in the thyroid.

To the best of our knowledge, the reviews or meta-analyses conducted so far have focused on the importance of selected nutrients in Hashimoto’s disease, i.e., selenium, vitamin D, iodine, gluten, zinc, iron, and goitrogens [8,10,11,13,14,15,16,17,18,19,20,21,22,23,24,25,26,27]. However, current clinical evidence of the effect of the dietary factors on HT is still scanty and inconclusive. Therefore, the aim of this systematic review is to examine all the data available in the literature about the effects of nutritional/dietary intervention on the course of Hashimoto’s thyroiditis measured by hormone and anti-body levels and body weight status normalization.

## 2. Materials and Methods

### 2.1. Data Sources and Search Strategy

In this systematic review, international electronic databases: Scopus and PubMed were used. The articles were searched by title or abstract or controlled keywords (MeSH): “Hashimoto disease”, “minerals”, “vitamins”, “diet”, “nutrition therapy”, and “nutrients”. An asterisk (*) was applied to the keywords to denote a wildcard term. MeSH words were verified against the MeSH 2023 terms index available at [28]. To search for MeSH terms, the following filters were used: “FullWord”, “Exact Match”, and “All Terms” (Main Heading (Descriptor) Terms + Qualifier Terms + Supplementary Concept Record Fields). Finally, articles were searched using the formula presented in Table 1. Studies potentially eligible for inclusion and available as of 30 November 2022 were identified.

### 2.2. Inclusion and Exclusion Criteria

The following inclusion criteria were used:

- original articles focusing on nutritional/dietary interventions (or nutritional/dietary interventions + supplementation) in patients diagnosed with Hashimoto’s disease;
- articles in English;
- articles to which full access has been granted.

The exclusion criteria included the following:

- articles other than original (case study, review, meta-analysis, commentary, book chapter, post-conference materials, and so on);
- articles that do not include dietary interventions;
- nutritional interventions without a control group;
- non-human studies;
- articles not focused on Hashimoto’s disease;
- articles including patients with thyroidectomy;
- supplementation-only interventions;
- interventions that were designed to induce Hashimoto’s disease;
- articles to which full access has not been granted despite attempts to contact a corresponding author.

### 2.3. Construction of the Review

This systematic review was prepared according to the PRISMA checklist [29] and the PICO model (P—population/patient; I—intervention/indicator; C—comparative/control; O—outcome):

- Population: Patients diagnosed with Hashimoto’s disease at any stage of life and regardless of gender and levothyroxine treatment status.
- Intervention: Nutritional/dietary interventions (healthy eating, elimination diets) or nutrition + supplementation.
- Comparison: The control group with/no nutritional/dietary intervention and with/no supplementation.
- Outcome: Parameters’ evaluation including at least one marker from the following: thyroid hormones (fT3, fT4); TSH; antibodies: anti-TPO and anti-TG; ultrasound examination of the thyroid gland; quality of life; anthropometric measurements (body weight, waist circumference, hip circumference); or body composition analysis [30].

All data were obtained from article texts, tables, and figures. The following information was searched for and extracted from the included articles: authors, year of publication, number of patients, sex of patients, Hashimoto’s disease diagnosis before inclusion to the intervention, the number of dropouts during the study, thyroid function (e.g., hypothyroidism, euthyrosis), characteristics of patients, criteria for inclusion and exclusion from the study, frequency of contacts during the intervention, characteristics and duration of nutritional intervention, markers in baseline and after the regiment, and type of study.

## 3. Results

### 3.1. Characteristics of Selected Articles

**Figure 1:**
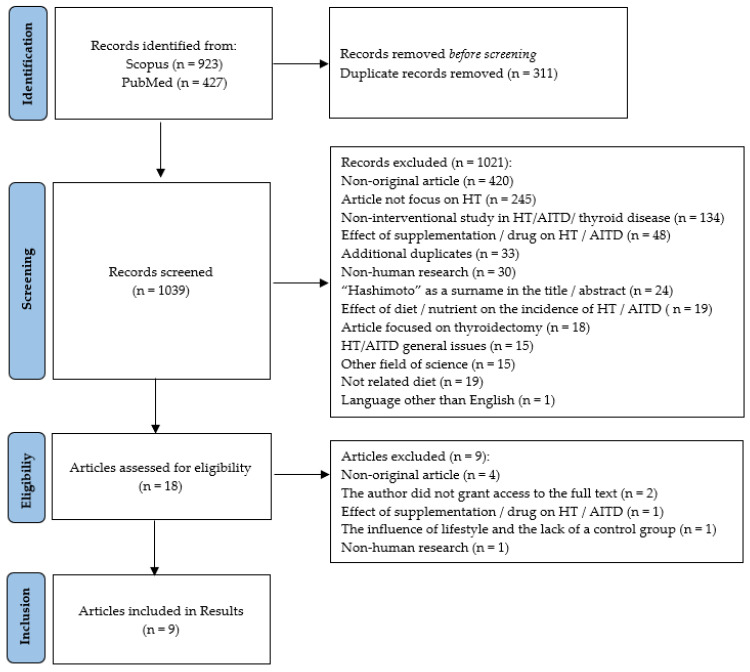
PRISMA flow diagram of the selected and included articles. HT—Hashimoto’s thyroiditis; AITD—autoimmune thyroid disease.

A total of 1350 studies were identified through two electronic database searches. After removing duplicates (*n* = 311), articles were reviewed by title or abstract by two independent researchers. Based on the inclusion and exclusion criteria, 18 articles were pre-qualified for the full text reading. Subsequent papers were excluded at this stage for reasons such as non-original article; the correspondence author did not grant access to the full text; effect of supplementation/drug on HT/AITD; the influence of lifestyle and the lack of a control group; and non-human research. During the selection of articles, the researchers discussed any ambiguous situations with each other and the classification of articles based on the inclusion and exclusion criteria. Finally, nine publications met the inclusion criteria; the detailed procedure is presented in Figure 1.

The included articles were published mainly during the last eight years [31,32,33,34,35,36,37,38], except for the one published in 2003 [39]. Four out of nine studies were conducted in Poland [31,32,33,36]. The other four studies were conducted in Turkey [38], Iran [34,35], Italy [37], and South Korea [39]. Patients were mainly recruited from clinics [32,34,35,38]. In the case of four studies [31,36,37,39], this information was not provided by the authors.

Four out of nine studies involved women only [31,32,33,36]. Hashimoto’s thyroiditis was diagnosed mostly on the basis of characteristic ultrasound features [31,32,33,36,38] and increased levels of any of the thyroid antibodies [31,32,33,36,37,38,39]. In two studies, the authors stated that HT was diagnosed by a licensed physician [34,35].

The main characteristics of the included studies and clinicopathologic features are presented in Table 2.

### 3.2. Characteristics of the Nutritional Intervention 

The details of nutritional interventions are described in Table 3.

Three studies involved a gluten-free diet [31,33,36] and two studies involved a restriction of selected food products [32,37]. Other interventions included the following: lactose elimination [38], iodine restriction [39], and black cumin consumption [34,35]. Intervention time ranged from 3 weeks [37] to 12 months [31,33]. The size of the experimental groups ranged from 16 [36] to 108 patients [37], while the control groups consisted of 12 [38] up to 72 individuals [37].

The lactose-free diet intervention included lactose-intolerant patients (euthyroid and with subclinical hypothyroidism) as an experimental group and euthyroid patients without lactose intolerance as controls [38]. In three studies, participants had normal thyroid function [31,33,36], whereas in the dietary iodine restriction intervention, participants were hypothyroid [39]. In four studies, this information was missing [32,34,35,37].

Patients were treated with levothyroxine in five out of nine studies [32,33,34,35,38]. In three studies, there was no such pharmacological treatment [31,36,39] and, in one study, information on pharmacological treatment was not provided [37].

The included studies differed in terms of dropout rates: 37% on lactose-free diet [38]; 33% on gluten-free diet [31]; and 15% on *Nigella stativa* intervention [34,35] and the reduction diet with/without the IgG test, including zinc and selenium supplementation [32]. In the case of the 6-month gluten-free intervention [36] and iodine restriction [39], all participants completed the study. Information on dropout rates is missing in the low-carbohydrate diet intervention involving 180 patients [37].

The study participants were contacted by the researchers weekly [34,35,37], every two months [31,32,36], or every three months [33]. In two studies, the evaluation was conducted at the baseline and repeated at the end of the intervention [38,39].

### 3.3. Main Outcomes

Krysiak et al. [31] examined the effect of a gluten-free diet on the concentration of vitamin D in women with Hashimoto’s disease. The experimental group was also diagnosed with non-celiac gluten sensitivity and recommended a gluten-free diet. The control group, without non-celiac gluten sensitivity, followed a regular diet. Both groups received the same dose of vitamin D (supplementation). In the control group, there was a significant decrease in anti-TPO (*p* = 0.0017) and anti-TG (*p* = 0.0056) levels and an increase in vitamin D concentration (*p* = 0.0006) compared with the experimental group. Although the typical diet, compared with the gluten-free diet, showed better results in improving the concentration of anti-TPO, anti-TG and vitamin D, in both groups, a significant improvement in these parameters was observed. However, no changes were observed in the case of TSH, fT3, and fT4 in the groups and between them. According to the authors, a gluten-free diet may hinder the absorption of vitamin D into the blood and thus hinder therapeutic support in lowering thyroid antibodies.

Ostrowska et al. [32] observed that body weight, BMI, and % body fat decreased significantly in both the experimental group (reduction diet + elimination products based on IgG test) (all *p* < 0.001) and the control group (reduction diet) (all *p* < 0.001). The experimental group showed a significantly better improvement in the aforementioned parameters than the control groups (*p* < 0.001, *p* < 0.002, and *p* < 0.026, respectively). TSH, fT3, fT4, anti-TG, and anti-TPO levels decreased significantly in both the experimental group (all *p* < 0.001) and the control group (all *p* < 0.001). However, the experimental group had a significantly better improvement in the previously mentioned parameters compared with the control groups (*p* < 0.001, *p* < 0.001, *p* < 0.001, *p* < 0.048, and *p* < 0.001, respectively). Additionally, the authors noticed that a decrease in BMI correlated with an increase in fT3 (*p* < 0.001) and fT4 (*p* = 0.003). The reduction in adipose tissue correlated with an increase in fT3 (*p* < 0.001) and fT4 (*p* = 0.035). The change in body weight, BMI, and adipose tissue content was positively correlated with anti-TG (*p* = 0.001, *p* < 0.001, *p* = 0.003 respectively). BMI change positive correlation with anti-TPO titer (*p* = 0.023).

A 12-month study by Pobłocki et al. [33] found that, in LT4-treated patients, a gluten-free diet decreased TSH (*p* < 0.044), but not thyroid hormones or anti-TPO and anti-TG antibodies. However, after analyzing the changes in the median concentration of the tested blood indices, a significance was noticed in TSH (*p* = 0.039) and fT4 (*p* = 0.022). An analysis of changes in the concentration of the studied parameters after logarithmic transformation was also performed, which showed the improvement in anti-TG, TSH, and fT4 at 3, 6, and 12 months of the intervention. A reduction in anti-TPO was also observed, but only in the third month of the trial.

During the consumption of 2 g *Nigella sativa* [34,35], a decrease in TSH (*p* = 0.03), weight (*p* = 0.004), BMI (*p* = 0.002), waist circumference (*p* = 0.006), and hip circumference (*p* = 0.001) was observed in levothyroxine-treated patients. In the experimental group, there was also an increase in T3 (*p* = 0.008) and improvement in some markers of oxidative stress and endothelial dysfunction. The concentration of anti-TPO, T4, nesfatin-1, and vascular endothelial growth factor (VEGF) remained unchanged. No significant changes were noticed in the control group. After the intervention, there was a significant improvement in the study group compared with the control group in terms of TSH (*p* = 0.02), T4 (*p* = 0.04), and anti-TPO (*p* = 0.01). In the experimental group, there was also an improvement in HDL cholesterol (*p* = 0.046), LDL (*p* = 0.002), and TG (*p* = 0.02), but not total cholesterol. However, there was a significant difference in HDL levels (*p* = 0.027) between the groups after the intervention. However, after the intervention, there was a significant improvement in HDL levels (*p* = 0.027) in the study group compared with the control group.

Krysiak et al. [36] investigated the effect of a 6-month gluten elimination diet on euthyroid untreated patients with Hashimoto’s disease. Patients also had positive anti-tissue transglutaminase antibodies (anti-tTG), but no clinical symptoms of celiac disease or diagnosed celiac disease. The gluten-free diet significantly reduced the anti-TPO and anti-TG antibodies in the experimental group compared with the control group (without a gluten-free diet). There was also an improvement in vitamin D level, which, according to the authors, could also have had an impact on the improvement in antibody titers.

The study by Esposito et al. [37] based on a low-carbohydrate diet with the exclusion of several products did not affect TSH, fT3, and fT4. On the other hand, it resulted in a decrease in BMI (*p* < 0.000), lean mass (*p* < 0.000), body weight (*p* < 0.000), fat mass (*p* < 0.05), anti-TG (*p* < 0.013), anti-microsomal (*p* < 0.000), and anti-TPO (*p* < 0.029). However, this intervention was much shorter than those used in other studies (3 weeks), making it difficult to compare results.

Asik et al. [38] examined the effect of an 8-week lactose-free diet on the course in levothyroxine-treated patients with Hashimoto’s disease. They observed that, after the intervention in the group of patients with HT and lactose intolerance, the TSH value decreased significantly (*p* < 0.05) compared with the results before the intervention. However, in the group of lactose-tolerant patients with HT, the results before and after were similar (*p* > 0.05). No significant change in fT4 was observed in any of the groups, which the authors explained with a too short intervention to draw robust conclusions. The authors also suggested that, in the case of a high dose of levothyroxine used by patients or resistance to its treatment and difficulty in regulating TSH, it is worth considering testing for lactose intolerance.

In the study by Yoon et al. [39], 18 of 23 patients (78.3%) in the experimental group had returned to normal thyroid function and 17.3% of patients had exacerbated hypothyroidism after 3 months. There was an improvement in fT4 and TSH (all *p* < 0.05) but not in T3 and T4 levels. In the control group, 10 out of 22 patients (45.5%) recovered to euthyroidism, 2 patients had hypothyroidism, and another 10 patients had an exacerbation of hypothyroidism. There was an improvement in fT4 (all *p* < 0.05) but not in T3, T4, and TSH levels. It is worth noting that this was a region with excessive iodine intake.

## 4. Discussion

Despite the small number of studies, the collected data demonstrate the potential positive effect of the nutritional strategies on the course of Hashimoto’s thyroiditis. Most often, nutritional intervention included the elimination of components such as gluten, lactose, or selected food products [32,33,36,37,38]. Among 83 patients, almost 76% of HT patients taking levothyroxine (LT4) were lactose-intolerant [38]. Lactose is a common component of levothyroxine formulations, which can lead to impaired LT4 efficacy in sensitive individuals [40]. Lactose intolerance is associated, among others, with bacterial overgrowth, malabsorption, and damage to the intestinal villi, which results in a greater demand for LT4. In the case of a need for a high dose of levothyroxine or resistance to treatment and difficulty in regulating TSH, it is worth considering testing patients for lactose intolerance [38]. The need for a higher dose of levothyroxine in lactose intolerant patients was reported by other authors [41]. However, Marabotto et al. [42] did not observe any differences in cumulative LT4 dose requirements in Hashimoto’s disease patients with or without lactose intolerance. To the best of our knowledge, studies to date have shown no benefit from lactose restriction in people with Hashimoto’s disease who have not been treated with levothyroxine.

The elimination of gluten from a diet is a treatment for diseases such as celiac disease, Duhring’s disease, wheat allergy, or non-celiac gluten sensitivity (NCGS) [43]. Among patients with Hashimoto’s disease, celiac disease is more prevalent [44]. This may result in a higher levothyroxine requirement owing to the lower absorption capacity of LT4 in the gastrointestinal tract [45]. Patients with Hashimoto’s thyroiditis may develop NCGS, which may be immune-related. It is not associated with celiac disease or allergies to wheat or gluten. It causes non-specific symptoms after eating foods containing gluten, such as headaches, joint and muscle aches, or the so-called “foggy mind”. Such symptoms often accompany Hashimoto’s disease [46]. According to a systematic review of a gluten-free diet on parameters in the course of Hashimoto’s thyroiditis, among the six included studies, no significant change in TSH was noted at any time [47]. In the case of anti-TPO and anti-TG, Krysiak et al. [36] observed a lowering of the antibody level and, in study by Valentino [48], one patient had a significantly reduced anti-TPO after 18 months (*p* < 0.001). According to the experts of the Polish Society of Parenteral Nutrition, Enteral Nutrition, and Metabolism (POLSPEN), the elimination of gluten from the diet in Hashimoto’s thyroiditis is unjustified, if there are no medical indications [49].

The meta-analysis of Song et al. [50] showed that obesity was correlated with Hashimoto’s disease (*p* = 0.022). A significant association was also observed between elevated anti-TPO (*p* = 0.001) and obesity, but no relationship was found between a positive anti-TG result and obesity. According to three meta-analyses, selenium supplementation leads to a reduction in antibodies, although, as the authors indicate, the quality of the included evidence is low [13,14,21]. According to the meta-analyses, *Nigella sativa* reduces body weight [51,52]. A systematic review by Khabbazi [53] suggested that *Nigella sativa* might be effective in the treatment of rheumatoid arthritis, especially in animal and in vitro studies, as well as in humans (a decrease in the disease activity score 28 (DAS28) was especially noted). It is also supposed to alleviate the course of other autoimmune diseases, such as autoimmune encephalomyelitis [54], ulcerative colitis [55], and psoriasis [56].

Iodine deficiency is a known factor in causing the goiter of the thyroid gland. To prevent iodine deficiency, fortifications have been introduced, e.g., with iodized salt. On the other hand, of consumption >1100 mcg/day can also lead to a thyroid dysfunction. Excess iodine is toxic to the thyroid cells because it causes the inflammation that leads to the development of Hashimoto’s thyroiditis [18]. Excess iodine may lead to the Wolff–Chaikoff effect, i.e., a temporary or permanent decrease in the synthesis of thyroid hormones and indirect inhibition of thyroid peroxidase activity [57]. However, the data from Denmark do not confirm the adverse effect of iodine fortification of food on the course of thyroid diseases [58].

### Strengths and Limitations

In the literature, there are still insufficient data indicating which nutritional/dietary interventions in patients with Hashimoto’s disease contribute to success in the context of metabolic parameters and body weight management. What sets this review apart is that it summarizes the results of nutritional interventions with a control group among patients with Hashimoto’s disease. Observational studies (cross-sectional studies, case reports, and animal studies) were excluded in order to obtain more reliable results. There was no time limit for searching for articles. The strength is the analysis of the results for an age homogeneous group—in this review, only adults were included. However, the studied group was not homogeneous with respect to the levothyroxine treatment, thyroid status, and outcomes evaluated.

However, this review is not without limitations. It is difficult to unequivocally deduce the role of nutritional intervention on the described parameters in Hashimoto’s disease for several reasons. First of all, the number of studies is small, the interventions are diverse, and other factors may have influenced the results (e.g., vitamin D). Secondly, the disease is not a homogeneous condition, and variability between patients is high. The basic supply of critical nutrients such as iodine, selenium, or iron has not been addressed in the studies and may differ between countries and individuals. For example, adequate iodine intake is reported for Poland, Turkey, Iran, and Italy, as well as excessive iodine intake for South Korea [59]. For this reason, it is likely that different populations will respond differently to the nutritional interventions analyzed, and recommendations may need to be given differently to patients residing in different areas of the world, where the baseline supply differs. Additionally, articles available only in two online databases and only published in English were analyzed. This search strategy eliminates publications in local languages as well as publications in paper-only journals.

## 5. Conclusions

Based on this systematic review, it is difficult to present unequivocal conclusions owing to the variety of nutritional support implemented, its duration, and the size of the groups. Previous studies have had a positive or neutral impact on biochemical parameters or symptoms in the course of Hashimoto’s thyroiditis to varying degrees. However, there is a great need for further research to clearly determine which type of nutritional intervention would be the most beneficial for patients with Hashimoto’s thyroiditis.

## Figures and Tables

**Table 1:** Strategy for the primary literature search conducted in PubMed and Scopus databases.

**Scopus (*n* = 923)**
(TITLE-ABS(“Hashimoto*”) OR KEY(“Hashimoto disease”)) AND (KEY(“diet”) OR KEY(“nutrition therapy”) OR KEY(“minerals”) OR KEY(“vitamins”) OR KEY(“nutrients”) OR TITLE-ABS(“diet*”) OR (TITLE-ABS(“nutrit*”) AND TI-TLE-ABS(“therap*”)) OR TITLE-ABS(“mineral*”) OR TITLE-ABS(“vitamin*”) OR TI-TLE-ABS(“nutrient*”))
**PubMed (*n* = 427)**
(“diet”[MeSH Terms] OR “nutrition therapy”[MeSH Terms] OR “minerals”[MeSH Terms] OR “vitamins”[MeSH Terms] OR “nutrients”[MeSH Terms] OR “di-et*”[Title/Abstract] OR (“nutrit*”[Title/Abstract] AND “therap*”[Title/Abstract]) OR “mineral*”[Title/Abstract] OR “vitamin*”[Title/Abstract] OR “nutrient*” [Ti-tle/Abstract]) AND (“Hashimoto*”[Title/Abstract] OR “Hashimoto Disease”[MeSH Terms])

**Table 2:** The characteristics of the included studies, summary of dietary interventions, and clinicopathologic features.

Authors, Year, Country	N, % Women, Age (Mean ± SD), BMI (Mean ± SD), % of Euthyroid Patients, % of Patients Taking LT4	Duration	Description of the Nutritional Intervention	Results
Krysiak et al., 2022, Poland [31]	EG (with non-celiac gluten sensitivity): 31, 100%, 35 ± 7, 22.6 ± 3, 100%, 0% CG (without non-celiac gluten sensitivity): 31, 100%, 36 ± 7, 23.5 ± 3.5, 100%, 0%, 0%	12 months	EG: Gluten-free diet + vitamin D (100 µg [4000 IU]/day) CG: Gluten diet + vitamin D (100 µg [4000 IU]/day)	↑ Anti-TPO (*p* < 0.0017), anti-TG (*p* < 0.0056) <-> TSH, fT4, fT3, fT3/fT4 ratio
Ostrowska et al., 2021, Poland [32]	EG: 45, 100%, 42.74 ± 10.51, 35.63 ± 4.06, nd, 100% CG: 40, 100%, 41.02 ± 11.96, 35.87 ± 5.59, nd, 100%	6 months	EG (reducing + elimination): Deficit at the level of 1400–1600 kcal + dietary elimination based on the IgG test + 200 mcg of 1-selenomethionine/day and 30 mg of zinc gluconate/day CG (reducing): Deficit at the level of 1400–1600 kcal + 200 mcg of 1-selenomethionine/day and 30 mg of zinc gluconate/day	↓ Weight (*p* < 0.001), BMI (*p* < 0.002), % body fat (*p* = 0.026), TSH (*p* < 0.001), anti-TPO (*p* < 0.001), anti-TG (*p* < 0.048) ↑ fT3 (*p* < 0.001) and fT4 (*p* < 0.001)
Pobłocki et al., 2021, Poland [33]	EG: 31, 100%, 36.64 ± nd, 26.27 ± nd, 100%, 100% CG: 31, 100%, 37.07 ± nd, 24.53 ± nd, 100%, 100%	12 months	EG: Gluten-free diet CG: Gluten diet	↓ TSH (*p* = 0.044) <-> fT3, fT4, anti-TPO, anti-TG
Farhangi et al., 2020 [34]; Farhangi et al., 2016, Iran [35]	EG: 20, 85%, 35.70 ± 8.18, 27.10 ± 4.63, nd, 100% CG: 20, 85%, 33.95 ± 8.72, 25.93 ± 4.07, nd, 100%	8 weeks	EG: *Nigella sativa* powder 2 g/day (1 g before lunch and 1 g before dinner) CG: Placebo-starch powder 2 g/day (1 g before lunch and 1 g before dinner)	↓ TSH (*p* = 0.02), anti-TPO (*p* = 0.01) ↑ T4 (*p* = 0.04) <-> weight, BMI, WHR, T3
Krysiak et al., 2018, Poland [36]	EG: 16, 100%, 30 ± 5, 22.9 ± 2.3, 100%, 0% CG: 18, 100%, 31 ± 6, 23.1 ± 2.1, 100%, 0%	6 months	EG: Gluten-free diet CG: Gluten diet	↓ Anti-TPO, anti-TG (*p* < 0.05) <->TSH, fT3, fT4
Esposito et al., 2016, Italy [37]	EG: 108, 50%, nd, nd, nd, nd CG: 72, 44%, nd, nd, nd, nd	3 weeks	EG: Diet based on the proportions of macronutrients: proteins 50–60%, fats 25–30%, carbohydrates 12–15%. Additional mandatory recommendations: eat vegetables, including large leafy vegetables, and only lean parts of white and red meat. Products such as eggs, dairy products, legumes, fruit, bread, pasta, goitre food, and rice were excluded CG: Low-energy diet with no exclusions as to the type of food consumed, but the patient should follow the recommended diet, according to the assumptions of the National Food and Nutrition Research Institute	↓ Weight (*p* < 0.05) and body fat mass (*p* < 0.05) (in the groups)
Asik et al., 2014, Turkey [38]	EG: 38 of LI, 97%, Age: E 45.67 ± 10.28, SCH 35.5 ± 9.87, 79%, 100% BMI: E 27.54 ± 5.77, SCH 30.34 ± 4.59 CG: 12 E without LI, 92%, 47.9 ± 8.73, 29.27 ± 3.67, 100%, 100%	8 weeks	EG: Lactose-free diet CG: Lactose-free diet	↓ TSH (*p* < 0.05) <-> fT4
Yoon et al., 2003, South Korea [39]	EG: 23, 100%, 40.70 ± 10.49, nd, 0%, 0% CG: 22, 86%, 43.50 ± 11.88, nd, 0%, 0%	3 months	EG: Limiting iodine intake with the diet to 100 mcg per day in a region with excessive iodine intakes CG: No restriction of iodine intake with the diet in a region with excessive iodine intakes	No investigated correlations between groups

LI—lactose intolerance, EG—experimental group, CG—control group, BMI—body mass index, E—euthyrosis, SCH—subclinical hypothyroidism, nd—no data, IgG—immunoglobulin G, TSH—thyrotropin, anti-TPO—antibodies against peroxidase, anti-TG—antibodies against thyroglobulin, T3—triiodothyronine, T4—thyroxine, fT3—free triiodothyronine, fT4—free thyroxine, LT4—levothyroxine, WHR—waist-to-hip ratio; ↑ —result higher in the study group than in the control group; ↓—lower result in the study group than in the control group; <-> —the result did not differ between the groups.

**Table 3:** Description of the implemented nutritional/dietary interventions.

Authors, Year, Country	Description of the Implemented Nutritional Support	
	Experimental Group	Control Group
Krysiak et al., 2022, Poland [31]	Avoiding cereal products containing gluten. As gluten-free substitutes, the researchers recommended eating foods produced by certified gluten-free food producers; Supplementation: vitamin D 100 µg [4000 IU] (once a day in the morning)	Remained on a gluten diet typical for the examined women; Supplementation: vitamin D 100 µg [4000 IU] (once a day in the morning)
Ostrowska et al., 2021, Poland [32]	Individual elimination of products, in accordance with the results obtained from the laboratory tests of food sensitivity type III in the immunoglobulin class G 1–3 by ELISA method; Macronutrient content in the diet—25% protein, 30% fat, 45% carbohydrates; The diet was balanced in terms of the standards for the demand for micro and macro elements for a given age group; Energy supply was at the level of 1400–1600 kcal (deficit of about 1000 kcal/day, depending on the individual resting metabolism and energy expenditure); Supplementation: 200 mcg of 1-selenomethionine/day and 30 mg of zinc gluconate/day.	Macronutrient content in the diet—25% protein, 30% fat, 45% carbohydrates; The diet covered the daily requirement for micro and macro elements for a given age group; Energy supply of 1400–1600 kcal (deficit of about 1000 kcal/day, depending on the individual resting metabolic rate and energy expenditure); Supplementation: 200 mcg of 1-selenomethionine/day and 30 mg of zinc gluconate/day.
Pobłocki et al., 2021, Poland [33]	The group received a freely available brochure on the gluten-free diet; Gluten-free diet was defined as the consumption of gluten-free natural and processed products containing ≤20 mg gluten/1 kg; All participants received a sample of a gluten-free diet menu; Education on the following: proper distribution of meals, energy during the day and the correct structure of fatty acid consumption, increased supply of omega-3 fatty acids and reduced consumption of saturated and trans fatty acids, products with a low glycemic index, a reduction in the supply of simple carbohydrates, an increased consumption of fiber.	A diet containing gluten, typical for the Polish population.
Farhangi et al., 2020 [34]; Farhangi et al., 2016, Iran [35]	Capsules with *Nigella sativa* powder 2 g/day (1 g before lunch and 1 g before dinner).	Capsules with placebo-starch powder 2 g/day (1 g before lunch and 1 g before dinner).
Krysiak et al., 2018, Poland [36]	Education by a medical doctor and dietitian; gluten-free diet leaflets.	A diet containing gluten, with no additional dietary recommendations.
Esposito et al., 2016, Italy [37]	Diet based on the proportions of macronutrients: proteins 50–60%, fats 25–30%, carbohydrates 12–15%; Eat vegetables, including large leafy vegetables and only lean parts of white and red meat; Products such as eggs, dairy products, legumes, fruit, bread, pasta, goitrogens food, and rice are excluded.	Low-energy diet with no exclusions as to the type of food consumed, but the patient should follow the recommended diet, in accordance with the assumptions of the National Research Institute on Food and Nutrition.
Asik et al., 2014, Turkey [38]	Reduction in amount of lactose in the diet (avoiding milk and dairy products, including skimmed milk powder and whey powder); Avoiding, especially in the morning, beverages (grapefruit juice, coffee) and foods rich in fiber and soybean meal.	Reduction of the consumption of these foods as the study group, especially in the morning; Avoiding, especially in the morning, beverages (grapefruit juice, coffee) and foods rich in fiber and soybean meal.
Yoon et al., 2003, South Korea [39]	Limiting iodine intake in the diet to 100 mcg per day.	No restriction of iodine intake in the diet.

## Data Availability

Not applicable.
